# A Multimethodological Approach for the Valorization of “Senatore Cappelli” Wheat Milling By-Products as a Source of Bioactive Compounds and Nutraceutical Activity

**DOI:** 10.3390/ijerph20065057

**Published:** 2023-03-13

**Authors:** Giuliana Vinci, Sabrina Antonia Prencipe, Federica Armeli, Rita Businaro

**Affiliations:** 1 Department of Management, Sapienza University of Rome, 00161 Rome, Italy; 2 Department of Medico-Surgical Sciences and Biotechnologies, Sapienza University of Rome, Corso della Repubblica 79, 04100 Latina, Italy

**Keywords:** wheat milling by-products, antioxidant activity, biogenic amines, microglia polarization, cytokines, anti-inflammatory activity, environmental sustainability, carbon footprint

## Abstract

Wheat is the third most cultivated cereal in the world and represents the major contributor to human nutrition. Milling wheat by-products such as husks (17–20% of the total processing output weight), even if still containing high-value-added bioactive compounds, are often left untreated or unused, thus resulting in environmental and human health burdens. In these regards, the present study is aimed at evaluating in a multimethodological approach the nutraceutical properties of durum wheat husks belonging to the ancient cultivar “Senatore Cappelli”, thus assessing their potential as bioactive compound sources in terms of phytochemical, cytotoxic, and nutraceutical properties. By means of HPLC-FD analyses, wheat husk samples analyzed revealed a higher content of serotonin, amounting to 35% of the total BAs, and were confirmed to occur at biogenic amines quality index (BAQI) values <10 mg/100 g. In addition, spectrophotometric assays showed a significant variable content in the phenolic (189.71–351.14 mg GAE/100 g) and antioxidant compounds (31.23–37.84 mg TE/100 g) within the wheat husk samples analyzed, according to the different cultivar areas of origin. Considering wheat husk extracts’ anti-inflammatory and antioxidant activity, in vitro analyses were performed on BV-2 murine microglia cells cultured in the presence or absence of LPS, thus evaluating their ability to promote microglia polarization towards an anti-inflammatory phenotype. Cytotoxicity assays showed that wheat extracts do not affect microglia viability. Wheat husks activity on microglial polarization was assessed by analyzing the expression of M1 and M2 markers’ mRNA by RT-PCR. Wheat husk antioxidant activity was assessed by analysis of NRF2 and SOD1 mRNA expression. Moreover, the sustainability assessment for the recovery of bioactive components from wheat by-products was carried out by applying the life cycle assessment (LCA) methodology using SimaPro v9.2.2. software.

## 1. Introduction

Cereals and cereal-based products represent one of the major components of human nutrition, thus representing the base of the food pyramid and accounting for more than 55% of the total consumption in the Mediterranean diet. Among cereals, durum wheat (*Triticum turgidum* L. subsp. *durum*) has a core role in the Italian diet and in the national economy, thus resulting in a production ranging between 3850 and 3900 million tons in 2021, with an increase of around +1.5% over 2020 [1].

Numerous studies have confirmed that cereals exert a protective action on human health, and they are a rich source of bioactive components, thus providing an excellent amount of dietary fiber, proteins, and antioxidants that can have health-promoting effects (i.e., cholesterol-lowering properties, anti-inflammatory effects, chronic diseases prevention, etc.) [2]. In particular, the reasons for the protective effects of cereals on human health could be mainly ascribed to the physio-chemical properties and structure of the grain (quantity, grain size, type of fiber, amount, and quality of phytochemical compounds as well as amylopectin and amylose content) [3]. Recent studies concerning the health benefits of wheat-based functional products have been increasingly focusing on the importance of introducing phytochemical compounds through the use of different wheat cultivars. Consequently, there is a renewed interest in the ancient varieties, particularly with regard to their potential nutraceutical quality [4].

During the milling process, most cereals, including wheat, undergo a series of treatments aimed at separating the outer fractions of the seed from the endosperm, intended for processing and transformation into cereal-based products (i.e., flour, pasta, bread, etc.). Nevertheless, increasing mechanization and industrialization have provided both food technologists and researchers with challenging problems arising from the production of processing by-products [5]. In particular, wheat husk (WH) is the major by-product of wheat milling. It is the outer layer of the grain, also called pericarp, that surrounds the endosperm and germ of the wheat grain. In whole wheat kernels, the WH is a multilayered tissue that accounts for 15–20% of the total processing weight, representing nowadays about 30 million tons of wheat milling by-products produced in the European Union [6]. Considering its physicochemical and organoleptic properties, WH consists of raw lignocellulosic material with a compact structure made up of cellulose (36–39%), hemicelluloses (18–21%), and lignin (16%) [7], which still contains a high content of bioactive compounds, particularly antioxidants such as phenolic compounds, carotenoids, etc. [7,8]. In these regards, different studies in the literature focus on the re-use of agricultural waste including wheat husks for renewable energy production [5,7], biofuel production, and biogas generation [9] as well as for the production of functional and value-added food products, cosmetics, feed for livestock use, natural bio-fertilizers, etc. [10]. 

Nevertheless, these residues or agro-industrial wastes are often left untreated or unused, so disposal is through dumping on land, incineration, or landfilling, thus resulting in the deposition of contaminants in the ecosystem and human health [11]. Therefore, the valorization of agricultural by-products through the extraction and recovery of molecules with high nutritional value (e.g., polyphenols, antioxidants, serotonin, etc.) as a new resource to be reused in other production processes could represent an alternative to incineration or composting. 

In these regards, the present study is aimed at evaluating in a multimethodological approach the nutraceutical properties of two ancient Italian durum wheat husks belonging to the ancient cultivar “Senatore Cappelli” from two different cultivation areas (Puglia and Tuscany), thus assessing their potential as bioactive compound sources in terms of phytochemical, antioxidant, and anti-inflammatory properties. To this purpose, the content of total phenolic compounds (TPC) and total flavonoid (TFC) and antioxidant activity were carried out by ABTS and DPPH assays.

Neurological disorders such as AD and PD are characterized by the accumulation of misfolded proteins that contribute to chronic microglial hyperactivation. The release of pro-inflammatory mediators that trigger neuroinflammation, exacerbated by oxidative stress, leads to neuronal death [12]. Much attention is now focused on bioactive molecules present in functional foods and in industrial processing waste for their anti-inflammatory and antioxidant properties to counteract neuroinflammation [13]. Considering that wheat husk extracts anti-inflammatory and antioxidant activity, in vitro analyses were performed on BV-2 murine microglia cells cultured in the presence/or absence of lipopolysaccharide (LPS), thus evaluating their ability to promote microglia polarization towards an anti-inflammatory phenotype. Neuroinflammation is the main driver of several chronic neurodegenerative diseases including Alzheimer’s disease (AD), Parkinson’s disease (PD), and major depression. Microglial cells possess the mechanisms to worsen the inflammation or on the contrary to lead to the repair of the damage depending on the stimuli they receive from the microenvironment [14]. When microglia cells are activated by an inflammatory stimulus, they take on a pro-inflammatory M1 phenotype associated with the expression of markers such as inducible nitric oxide synthase (iNOS) and cyclooxygenase-2 (COX-2) that mediate inflammatory signaling through Toll-like receptor 4 (TLR4) [15]. The M1 phenotype is also associated with the release of pro-inflammatory cytokines, interleukins, and chemokine ligands such as CCL2 [16]. 

When the injury resolves, the microglia polarizes toward an anti-inflammatory M2 phenotype and, with the release of anti-inflammatory cytokines associated with Arginase-1 (Arg-1) and expressed by macrophages, plays a key role in immune response regulation, primarily through the competition between intracellular iNOS and Arg-1 for arginine. M2-activated microglia upregulate the expression of another anti-inflammatory mediator, namely CD206, a mannose receptor pattern-recognition receptor [17]. An additional anti-inflammatory marker is chitinase-like 3 (Chil3), which encodes for the protein Ym1 [18]. To evaluate M1 or M2 states in microglial cells untreated and extract-treated in the absence or presence of LPS, we analyzed the mRNA levels of M1 and M2 markers. In addition, we investigated the antioxidant effect of wheat-husk-derived extracts by analyzing the mRNA expression of key genes involved in the cellular antioxidant system.

In addition, to evaluate the quality and safety of raw matrices, the detection of eight biogenic amines, namely 2-phenylethylamine (B-Pea), putrescine (Put), cadaverine (Cad), histamine (His), tyramine (Tyr), spermine (Spm), spermidine (Spd), and serotonin (Ser), was investigated by means of high-performance liquid chromatography coupled with fluorometric detection (HPLC-FD).

Moreover, the sustainability assessment for the recovery of bioactive compounds from wheat by-products was carried out through the application of the life cycle assessment (LCA) methodology by using SimaPro v9.2.2. software.

## 2. Materials and Methods

### 2.1. Chemicals

2-phenylethylamine (B-Pea), putrescine (Put), cadaverine (Cad), histamine (His), tyramine (Tyr), spermine (Spm), spermidine (Spm), serotonin (Ser), Dansyl-Chloride (DSN-Cl), sodium bicarbonate (NaHCO_3_), ammonium hydroxide (NH_4_OH), sodium hydroxide (NaOH), sodium carbonate (Na_2_CO_3_), Folin–Ciocalteu reagent (H_3_[P(W_3_O_10_)_4_]/H_3_[P(Mo_3_O_10_)_4_], 2,2-Diphenyl-1-picrylhydrazyl (DPPH), 2,2′-azino-bis (ABTS), sodium nitrite (NaNO_2_), and aluminum chloride (AlCl_3_) were purchased from Sigma-Aldrich (Milan, Italy). The following solvents were purchased from Sigma-Aldrich (St. Louis, MO, USA): acetone (C_3_H_6_O), perchloric acid (HClO_4_), acetonitrile, ACN (CH_3_CN), methanol (CH_3_;OH), and distilled water (d-H_2_O), all of which were HPLC-grade. Further used materials include Dulbecco’s Modified Eagle’s Medium High Glucose (DMEM, Sigma Aldrich, St. Louis, MO, USA); Fetal Bovine Serum (FBS, Sigma Aldrich, St. Louis, MO, USA); L-glutamine of penicillin-streptomycin, non-essential amino acids, and sodium pyruvate (Sigma Aldrich, St. Louis, MO, USA); 1× Tripsin-EDTA (Aurogene, Rome, Italy); Trypan Blue solution (1:1) (Corning, Glendale, AZ, USA); Lipopolysaccharide (LPS Sigma Aldrich, St. Louis, MO, USA); Qiazol Lysis Reagent (Qiagen, Hilden, Germany); and Power SYBR^®^ Green PCR Master Mix (Applied Biosystem, Foster City, CA, USA). RNA was extracted miRNeasy Micro kit (Qiagen, Hilden, Germany). The cDNA was generated using the High-Capacity cDNA Reverse Transcription Kit (Applied Biosystems, Foster City, CA, USA).

### 2.2. Instruments

The instruments used for the analyses were the following: NEYA 10R refrigerated centrifuge (Exacta Optech, Modena, Italy), IKA T18 digital Ultra–Turrax (IKA-group, Saufen, Germany), Bandelin Sonorex RK100H water and ultrasonic thermostatic bath, Whatman 0.45 µm 100 (PTFE) syringe filters (Sigma Aldrich, Milan, Italy), and UV–vis spectrophotometer (Jenway, Stone, UK). The chromatographic analysis of biogenic amines was performed using an ATVP LC-10 HPV binary pump with an RF-10° XL fluorometric (FD) detector (Shimadzu, Kyoto, Japan) working at λ emission = 320 nm and λ excitation = 523 nm. A Supelcosil LC-18 column (250 mm × 4.6 mm, 5 µm) with a Supelguard LC-18 (Supelco, Bellefonte, PA, USA) pre-column was used for the analysis of biogenic amines as well as a Steri-Cycle CO_2_ Incubator (Thermo Electron Corporation, Waltham, MA, USA). Cell culture 6-well and 48-well plates (Sarstedt, Nümbrecht, Germany) were used; RNA was quantified with NanoDrop One/OneC (Thermo Fisher Scientific, Waltham, MA, USA), and quantitative real-time PCR (qPCR) was performed on an Applied Biosystems 7900HT fast real-time PCR system (Applied Biosystem, Cheshire, UK) using the SDS2.1.1 program (Applied Biosystem, Foster City, CA, USA),

### 2.3. Sampling

**Figure 1:**
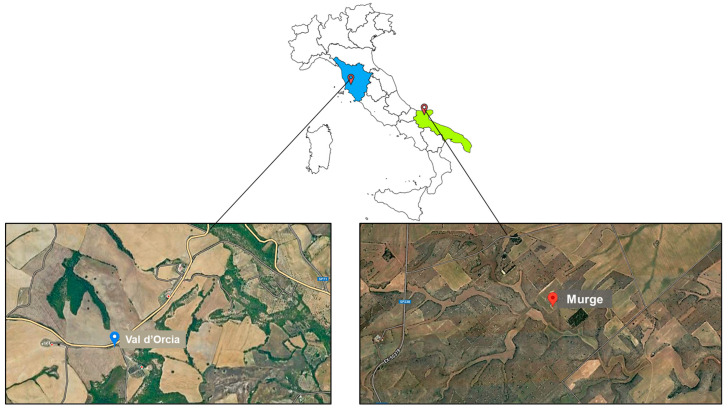
Geographical location and areas of origin for the wheat husk samples analyzed.

The study analyzed two wheat husk (WH) samples of the ancient “Senatore Cappelli” (SC) cultivar from two different wheat production chains. The first SC cultivar was located in the hilly territory of Val d’Orcia (WH1) in the Tuscany region, while the WH2 samples were cultivated on the karstic Murge upland, which is located in the Puglia region, as shown in Figure 1. In particular, owing to the peculiar pedo-climatic characteristics of these areas (mild climate, distributed rainfall throughout the year), the soil is characterized by a high percentage of loam and clay and a lower percentage of sand; it is also flat and has good drainage. Both samples were cultivated from October 2021 to June 2022. Approximately 200 g of each SC durum wheat husk was previously ground finely using a blender and then sieved using a sieve with 0.7 to 2.0 mm diameter holes. The obtained particle size fractions were collected and stored at refrigerated temperature, namely T = −18 °C, until the day of analysis. Raw matrices WH1 and WH2 had a moisture content of 6% when analyzed.

**Figure 2:**
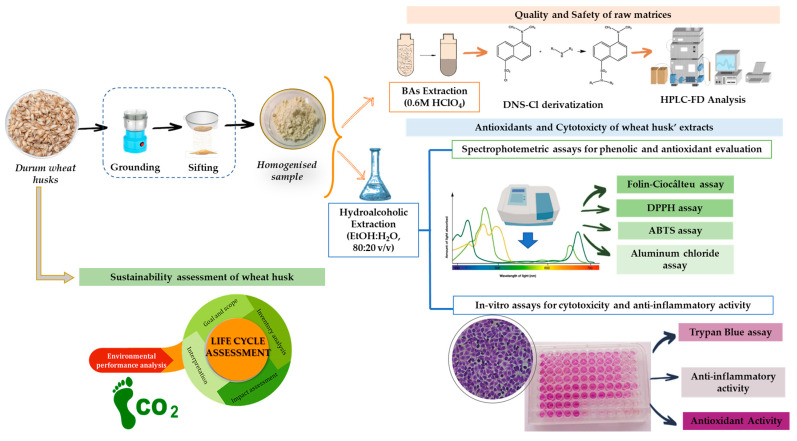
Multimethodological approach for the recovery of bioactive compounds from the wheat husk.

Both raw matrices and hydroalcoholic wheat husk extracts were characterized by HPLC chemical, spectrophotometric, anti-inflammatory, and cytotoxicity analysis, as shown in Figure 2.

### 2.4. Quality and Safety of Raw Matrices

#### Biogenic Amines Extraction from Wheat Husk

The biogenic amines (BAs) determination was carried out according to a previously published method [19] with some modifications. Briefly, 1 g (±0.01 g) of SC wheat husk was added with 12 mL of 0.6 M HClO_4_. The samples were then homogenized and centrifuged at 2700 rpm for 10 min at T = 25 °C. The supernatant was collected in a flask. The BAs extraction procedure was repeated twice. Then, the second extract was added to the first one and filtered through a 0.45 µm membrane syringe filter. The final volume was adjusted to 25 mL with 0.6 M HClO_4_. For the derivatization procedure, a 1 mL aliquot of the final extract was added to 200 µL of 2 M NaOH, 300 µL of saturated NaHCO_3_ solution, and 2 mL of dansyl chloride solution (10 mg/mL in acetone). After stirring, the samples were left in the dark for 60 min at 45 °C. To stop the dansyl chloride reaction, ~100 µL of 25% NH_4_OH was added. The final volume was made up to 5 mL by adding acetonitrile. The dansylated extract was filtered using a 0.45 µm filter and injected into the HPLC system. Analytes were eluted using Supelcosil LC-18 column (250 mm × 4.6 mm; 5 µm) in reverse phase with Supelguard LC-18 pre-column coupled with fluorometric detection. The flow rate was set at 1.2 mL/min, and the column temperature was set at T = 30 °C. The elution sequence started with 3 min of isocratic elution (50% ACN; 50% water), reaching 100% ACN after 18 min. Subsequently, the starting isocratic condition (50% ACN; 50% water) was restored. Method accuracy (recovery > 95%), and precision (RSD < 4.6%) were evaluated by analyzing the SC extracts at three different concentrations of BAs. The results obtained from the triplicate analysis were expressed through a calibration curve for each BA, ranging from 0.1 and 25 mg/L. The biogenic amines quality index (BAQI) was calculated based on BAs results to determine the SC samples’ quality loss. For BAQI values <10, the product can be considered safe [20]. It was calculated as follows and expressed in µg/g:

$$\mathrm{BAQI}\;=\frac{\left(\mathrm{PUT}+\mathrm{CAD}+\mathrm{HIS}\right)}{\left(1+\mathrm{SPM}+\mathrm{SPD}\right)}$$

### 2.5. Phytochemical, Cytotoxic, and Anti-Inflammatory Properties of Wheat Husk Extracts

#### 2.5.1. Hydroalcoholic Extraction of SC Wheat Husk

Sample extraction was performed according to the method of Zhang et al. (2021) with slight modifications [21]. About 10 g (±0.01 g) of each representative husk sample was weighted and placed into 50 mL glass centrifuge tubes, and 25 mL of ethanol in aqueous solution (80:20, *v*/*v*) was added. The samples were homogenized in an ultrasonic and thermostatic bath (Bandelin Sonorex, RK100H) at 400 MHz and at room temperature for 5 min and then centrifuged at 2700 rpm for 10 min at T = 25 °C with a NEYA 10R refrigerated centrifuge (Exacta Optech, Modena, Italy). The supernatant was collected in a 50 mL flask. The residue was added with 25 mL of ethanol in aqueous solution (80:20, *v*/*v*), mixed, and again centrifuged for 10 min. Then, the second extract was added to the first one and filled with ethanol/water (80:20, *v*/*v*) to the mark. For targeted analysis of “Senatore Cappelli” durum wheat husk samples (polyphenols, antioxidants anti-inflammatory activity), the extracts were filtered using 0.45 µm filter (Whatman^®^ Puradisc filters, Sigma Aldrich, Milan, Italy). Extractions were performed on the day of analysis, and extracts were stored at T = 4 ± 2 °C.

#### 2.5.2. Total Phenolic Content (TPC)

The total phenolic content of wheat husk samples was determined according to the Folin–Ciocâlteu method, as Gobbi et al. [19] reported. The absorbance of the samples was read at 750 nm against blank solution (EtOH:H_2_O, 80:20 *v*/*v*). The results were expressed as milligrams of gallic acid equivalents per g of wheat husk (mg GAE/g wheat husk). The results were obtained through a calibration curve ranging from 20 to 250 mg/L (R^2^ = 0.9998). All the measurements were carried out in triplicate. 

#### 2.5.3. Total Flavonoids Content (TFC)

The TFC of wheat husk samples was evaluated according to the aluminum-chloride method described by Abdel-Naeem et al. (2021), with some modifications [22]. To 0.5 mL of the hydroalcoholic extract, 2 mL of d-H_2_O and 150 µL of NaNO_2_ (5% *w*/*v*) were added to a 5 mL volumetric flask. The solution was stirred and incubated in the dark for 5 min, then 150 µL of AlCl_3_ (10% *w*/*v*) was added, and the solution was put back in the dark for 5 min. Next, 2 mL of NaOH (1 M) was added to the solution and left in the dark for a further 15 min. Subsequently, samples were made up to final volume of 5 mL by adding d-H_2_O. The absorbance of the wheat husk samples was read at 510 nm against EtOH:H_2_O, 80:20 *v*/*v*. TFC results were expressed as milligrams of rutin equivalent (RE) per g of the wheat husk (mg RE/g) by linear regression, ranging between 50 and 1000 mg/L (R^2^ = 0.9995). The results were expressed as means ± standard deviation (SD) of three replicates.

#### 2.5.4. Antioxidant Activity Determination by ABTS and DPPH Assays

The antioxidant activity of wheat husk samples was evaluated using two different spectrophotometric analyses: DPPH and Trolox-equivalent antioxidant capacity (TEAC) assays. The free radical scavenging activity of wheat husks was evaluated by the DPPH assay, according to a previously reported method [23]. The scavenging activity was measured at 517 nm. All experiments were assessed in triplicate, and values were reported as a mean of EC50 ± standard deviation (SD); the EC50 corresponded to the concentration of the wheat husk samples (mg/mL of extract) that provided 50% of the radical scavenging activity. Eight different concentrations of gallic acid diluted in methanol (100–1 mg/L) were prepared and used as a positive control. 

The TEAC of the wheat husk samples was estimated by the ABTS radical scavenging assay, according to Pierre et al. (2015) with slight modifications [24]. Briefly, 0.4 mL of wheat husk extract was added with 3.6 mL of ABTS radical solution and left in the dark for 15 min. ABTS radical decolorization was evaluated by measuring the absorbance at 734 nm. The results were expressed as milligrams of Trolox equivalent (TE) per g of wheat husk (mg TE/g) by a calibration curve ranging from 0.5 to 200 mg/TE (R^2^ = 0.9963).

#### 2.5.5. Trypan Blue Assay

The mouse microglia cell line (BV2) was seeded in DMEM containing 5% FBS, 1% L-glutamine, 1% penicillin-streptomycin, 1% non-essential amino acids, and 1% sodium pyruvate at 37 °C in a humidified atmosphere with 5% CO_2_. BV2 cells were arrayed in 48 wells (30,000 cells/400 µL) [25].

Treatment with the extracts obtained from the wheat husk (10, 50, and 100 ng/mL) was added, and the cells were incubated for 24 h at T = 37 °C. After 24 h, the cells were detached with Trypsin-EDTA 1× and counted through Burker’s chamber in Trypan Blue solution (1:1). Both live and dead cells were counted. 

#### 2.5.6. Real-Time Quantitative PCR Analysis

BV-2 cells were seeded onto 6-well plates at a density of 10^6^/well in 1 mL of DMEM. After a 45 min pre-treatment with extracts obtained from husks at concentrations of 10 and 100 ng/mL, cells were added with LPS 1 ng/mL and incubated at 37 °C for 4 h. The inflammatory stimulus was added with LPS 1 ng/mL for 4 h. After 4 h, cells were detached in 700 µL of Qiazol Lysis Reagent and stored at −80 °C. RNA was extracted and quantified from BV-2 cells. The cDNA was originated using the High-Capacity cDNA Reverse Transcription Kit. Quantitative real-time PCR (qPCR) was performed for each sample in triplicate on an Applied Biosystems 7900HT fast real-time PCR using the Power SYBR^®^ Green PCR Master Mix. Primers for real-time PCR amplification were designed with UCSC GENOME BROWSER (http://genome.cse.ucsc.edu/ accessed on 11 January 2023); University of California, Santa Cruz) (Table 1). The comparative threshold cycle (CT) method was used to analyze the real-time PCR data. The target quantity, normalized with respect to the endogenous β-actin primer reference (ΔCT) and relative to the untreated control calibrator (ΔΔCT), was calculated using equation 2^−ΔΔCT^ [25].

### 2.6. Sustainability Evaluation of Wheat By-Products by Life Cycle Assessment (LCA)

The study evaluated the sustainability assessment of wheat by-products, which account for 17–20% of the total production, through the application of the life cycle assessment methodology in accordance with ISO 14040, 2006 and ISO 14044, 2006 [1]. SimaPro v9.2.2., software was used for the evaluation of environmental impacts. 

#### 2.6.1. Goal and Scope Definition

**Figure 3:**
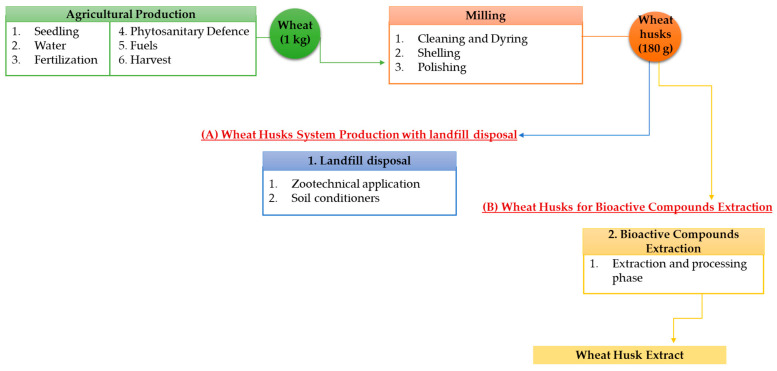
System Boundary of Durum Wheat Production considering (**A**) wheat husks for landfill disposal and (**B**) wheat husks for bioactive compounds extraction.

The goal of the study is to assess the environmental impacts associated with durum wheat by-products. In particular, the functional unit (FU) was identified as the production of 180 g of processing by-products (including husks) resulting from the milling process of 1 kg of wheat, thus making the assumption that this by-product corresponds to about 18% of the total wheat production according to a cradle-to-gate approach, as shown in Figure 3. The wheat husk system production includes the upstream process activities (i.e., seedling, water for irrigation, fuels, fertilization, etc.) for the agricultural production of 1 kg of wheat. Primary information was obtained through face-to-face interviews with farmers and mill owners based on the in-field activities for agricultural and milling phases. The milling phase includes the stages from cleaning to polishing, thus considering the use of water and electricity. In addition, secondary source data from the literature were used for the energy-production phase. In the first scenario, wheat husks are destined to landfill disposal as a unitary quantity of 180 g used for zootechnical application and soil conditioners. Otherwise, the wheat husks extraction scenario considered both the extraction and processing phase of wheat husks. 

#### 2.6.2. Life Cycle Inventory (LCI)

The unit processes of each phase were considered as well as the inputs from the agricultural phase to alternative scenarios for the extraction of bioactive compounds, thus considering the milling by-products generated. In particular, the inputs referred to a national average durum wheat production in the year 2020, thus focusing on the milling process of 1 kg of wheat; meanwhile, for the recovery of bioactive compounds from wheat milling by-products (husk), the inputs referred to the extraction process carried out on both samples, i.e., WH1 and WH2, analyzed in the study. Inputs for the wheat husk system production and recycling scenarios are shown in Table 2. All data referred to the same FU of 180 g of wheat husk resulting from the milling process of 1 kg of wheat, thus making the assumption that this by-product corresponds to about 18% of the total wheat production based on production estimations at national level [6]. LCI calculations were performed to model inputs for equipment, solvents, and electricity (i.e., ultrasonic bath, centrifugation, etc.) used during the extraction process. About 180 g of wheat husk was extracted for the recovery of bioactive compounds conventionally by using the hydroalcoholic solution of EtOH:H_2_O (80:20, *v*/*v*) as extractant.

*EcoInvent* version 3.8, *Agribalyse* v3.0.1, and *World Food LCA Database* (*WFLDB*) databases were used to calculate the environmental impacts of the extraction and processing phase of the wheat husks. 

#### 2.6.3. Scenario Analysis

To highlight the amount of CO_2_ avoided as a result of the possible reuse of the by-product and their environmental compatibility, two scenarios were proposed: (S1) relating to disposal of the by-product in the landfill and (S2) concerning the valorization of wheat by-products through the recovery of bioactive compounds.

The scenarios were subsequently compared through carbon footprint (CF) calculation. CF was calculated based on the LCI and LCIA results. CF is a measure expressing the greenhouse gas emissions (GHGs) caused by a product, service, or process. In accordance with the Kyoto protocol, CF is expressed in kilograms of CO_2_ equivalent (kg of CO_2_ eq), and it was calculated according to Forster et al. (2007) [26] based on Equation (1):

Carbon footprint = ∑G.G._i_ × k_i_ (1)

where G.G._i_ represents the amount of GHGs produced, and k_i_ corresponds to the CO_2_-equivalent coefficient for that gas. 

The CF was obtained employing the Green Gas Protocol V1.03/CO_2_ eq (kg) method (GHGP 2020) by using SimaPro v.9.2.2. software.

### 2.7. Statistical Analysis

The data were obtained from the analysis of three replicates and were expressed as mean ± standard deviation (SD) from experiments. The significance of differences between the extracts was tested using a one-way analysis of variance (ANOVA) with *p* < 0.05. After ANOVA, multiple comparison tests were performed for statistically significant variables, using Dann’s post hoc test (homogeneity of variance was assumed) at the level of *p* < 0.05. Statistical analyses were performed using unpaired Student’s *t*-test (GraphPad Software Inc., San Diego, CA, USA). 

## 3. Results and Discussion

### 3.1. Quality and Safety of Wheat Husks by Quantitative Determination of Biogenic Amines (BAs)

The content of eight BAs was evaluated in raw wheat husks by high-performance liquid chromatography with fluorescence detection (HPLC-FD). The analyzed wheat husk samples showed a variable content (*p* < 0.05) of total biogenic amines (Table 3).

In particular, WH2 samples showed a higher total BAs content (43.26 mg/100 g) than WH1 (35.66 mg/100 g) (*p*-value < 0.05). Thus, for both cases, SER is the most abundant biogenic amine, accounting for about 35% of the total content. This was in line with the literature results, which found an SER content ranging from 5.2 to 22 mg/100 g dw in wheat by-products [27]. Different authors reported that the occurrence of SER in plant-origin foods was affected by plant variety, degree of microbiologic contamination, and specific conditions to proliferate cells (pH, temperature, access to oxygen, etc.) [28]. Furthermore, it is well established that SER demonstrates positive effects on human health (psychoactive effects, vasoconstrictive properties, etc.); therefore, wheat husks relatively rich in SER could be of interest both to consumers and the food industry. It is relevant to underline BAs’ higher amounts of PUT, SPD, and SPM, detected in the highest amount in WH1 (1.81 ± 0.19 mg/100 g dw; 7.86 ± 0.81 mg/100 g dw; and 4.61 ± 0.47 mg/100 g dw, respectively). According to different authors [29], polyamines putrescine as well as spermine and spermidine are ubiquitous and endogenous in all plant-origin foods, and they have a relevant role in increasing food shelf life, thus representing a food-spoilage index [28,29]. In particular, the literature results highlighted PUT (0–8.6 mg/100 g), SPD (0–33 mg/100 g), SPM (0–4 mg/100 g), and SER (0–13 m/100 g) as the major BAs detected in durum wheat cultivars; their content in the outer layers of the grain (i.e., bran) is about 40–60% higher than in milling products such as whole and white flours [27]. Furthermore, the presence of exogenous monoamines CAD, HIS, and TYR detected in WH2 samples at a concentration of 2.1 ± 0.11 mg/100 g dw, 6.60 ± 0.79 mg/100 g dw, and 1.12 ± 0.09 mg/100 g dw, respectively, may be related to the storage and processing conditions of husks, thus representing a quality process marker. BAs such as HIS, CAD, and TYR are responsible for food-born illnesses such as Scombroid syndrome, cheese reaction, and food allergies, even if present at small concentrations [27]. The present results showed a variable content of these BAs among wheat milling by-products, but they were not as high as amounts reported for meat, fish, alcoholic beverages, cheese, and fermented vegetables (up to 1000–2000 mg/kg), thus representing the most involved foods in toxicological or allergic reactions. These trends are in line with the literature results reporting low BAs contents in cereals in comparison with the above-mentioned foods [27,28,29]. However, the biogenic amines quality index (BAQI) showed that the BAs amounts detected in wheat husk samples do not pose a health risk to the consumer since they presented values <10 mg/100 g [20].

### 3.2. Phenolic and Antioxidant Properties of “Senatore Cappelli” Durum Wheat Husks

During the wheat milling process, huge amounts of by-products are generated, accounting for 17–20% (*w*/*w*) of the total raw wheat. They generally consist of husks, outer germ layers, and bran, which still have biological and phytochemical properties such as polyphenols and antioxidants that could enhance health-promoting effects. 

The phenolic and antioxidant profile of SC wheat husks was evaluated by means of spectrophotometric assays, as shown in Table 4.

Results highlighted a variable content of phenolic and antioxidant compounds among husk samples depending on the area of origin. In particular, WH2 resulted in the highest total phenolic content (351.14 ± 5.91 mg/100 g dw), total flavonoids (156.90 ± 2.31 mg RE/100 g dw), and antioxidant activity considering the ABTS assay (37.84 ± 4.69 mg TE/100 g dw). Meanwhile, wheat husk samples from the Val d’Orcia wheat chain (WH1) showed a 40% (*p* < 0.05) lower phenolic and antioxidant potential than WH from the Puglia chain. However, the results of the DPPH assay differed from those of the ABTS assay; this may be probably attributed to the different types of radical agents used. The DPPH reagent, in fact, is a stable nitrogen radical that interacts mainly with peroxide radicals involved in lipid peroxidation, whereas the ABTS reacts with both hydrophilic and lipophilic radicals. Therefore, the reactivity of DPPH is only limited to the lipophilic fraction [19]. In particular, the different content of phenolic and antioxidants among wheat husk samples analyzed may be attributable to the varying pedo-climatic characteristics for the wheat’s area of origin despite belonging to the same cultivar (“Senatore Cappelli”). To this purpose, Dinelli et al. (2013) affirmed that bioactive compound content may greatly vary depending on durum wheat genotype as well as differences in genetic and agricultural crop management [3]. In these regards, it is well established that the biosynthesis and accumulation of phenolic compounds during kernel development is greatly influenced by the wheat variety, environmental conditions, as well as abiotic and biotic stresses [30]. 

Considering TPC values, most literature data reported similar trends in durum wheat by-products in terms of free soluble fraction, which accounts for 50–75% of the total amount of phenolics, thus highlighting flavonoids and phenolic acids as the most abundant in wheat milling by-products [31]. In these regards, considering TPC, values ranging from 80.90–610.49 mg GAE/g were found in durum wheat by-products [32] as well as values that ranged within 10.84–26.73 mg TE/g and 3.61–1194.8 mg/mL for ABTS assay [33]. Nevertheless, antioxidant activity by DPPH assay showed different trends strictly depending on the chemical composition of cereals; in particular, this result may be due to the lipophilic fraction contained in the wheat by-product, which may have contributed to the extract’s activity during the extraction process [33].

### 3.3. Cytotoxicity of “Senatore Cappelli” Durum Wheat Husks in BV2 Cells

**Figure 4:**
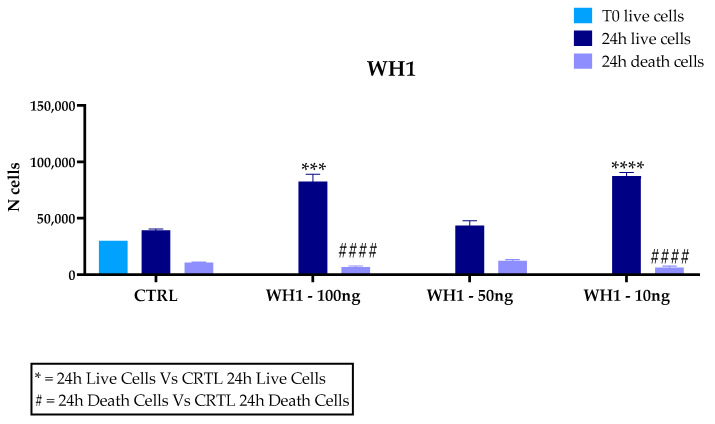

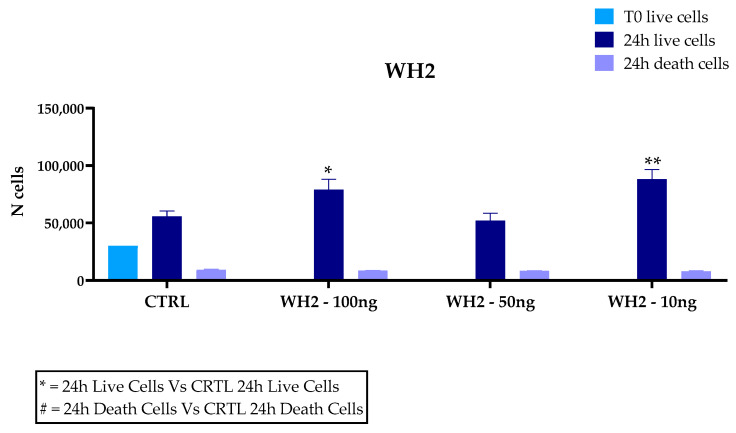
Trypan Blue assay for cytotoxicity of extracts on BV2 microglia cells. Cells were treated with 10, 50, or 100 ng/mL extracts of the husk (WH) of two supply chain. Data are reported as mean ± SD and normalized to the control of at least three independent experiments, and statistical analysis was reported using unpaired Student’s *t*-test. * *p* < 0.05; ** *p* < 0.01; *** *p* < 0.001; **** *p* < 0.0001; #### *p* < 0.0001.

Microglia play a key role in driving neuroinflammation, a mechanism underlying several neurodegenerative diseases; therefore, they represent a suitable model for investigating the anti-inflammatory and antioxidant activity of plant-derived bioactive molecules. Our results dealing with the cytotoxic activity of husk extracts on microglial cell cultures are depicted in Figure 4. The percentage of live cells in WH1 in the total number of cells at the concentration of 100 ng/mL is 92.6%; at the concentration of 50 ng/mL, it is 77.9% and at the concentration of 10 ng/mL is 93.2%. The percentage of live cells in the untreated cells (CTRL) out of the total corresponds to 78.64%. The percentage of live cells for WH2 is 90.2% at the concentration of 100 ng/mL; at 50 ng/mL, the percentage of live cells is 86.2% and at 10 ng/mL is 91.7%. The percentage of live cell control is 85.7%. Extracts obtained from husk, from the two different supply chains mentioned above, added to the in vitro cultures showed no toxicity at the concentrations of 100, 50, and 10 ng/mL. Indeed, BV2 cells treated with the husk extracts at 100 and 10 ng/mL showed a significantly higher proliferation compared to untreated cells (CTRL).

An increase in dead cells was observed in WH1 compared to controls, although there was an increase in cell number. At 50 ng/mL concentration, no significant differences with untreated cells were recorded in both WH1 and WH2. 

### 3.4. M1 mRNA Markers Expression

**Figure 5:**
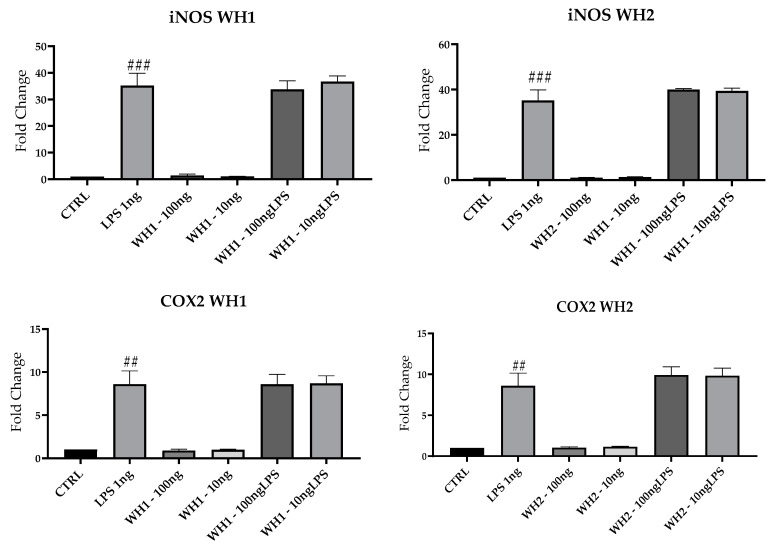

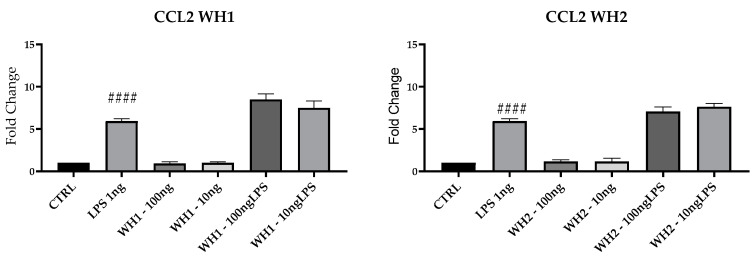
mRNA expression of iNOS, COX2, and CCL2 was evaluated by qRT-PCR. Data are shown as mean ± SD from three independent experiments performed in triplicate. Expression profiles were determined using the 2^−ΔΔCT^ method. # VS CTRL; ## *p* < 0.01; ### *p* < 0.001; #### *p* < 0.0001.

To evaluate the possible pro-inflammatory effect of the chaff-derived extracts of the two aforementioned dies, we analyzed the mRNAs of key M1 markers in BV2 cells. Results for mRNA expression of iNOS, COX2 and CCL2 mRNAs, and M1 markers highlighted a significant increase following LPS treatment compared with untreated cells (CTRL) (Figure 5).

Pretreatment with husk-derived extracts from both strands (WH1 and WH2) did not modulate iNOS, COX2, and CCL2 mRNAs expression. Husk extracts are devoid of any pro-inflammatory activity because they do not increase the expression of M1 markers.

Several randomized control trials have shown that the intake of whole grains compared to refined grains reduces the expression of pro-inflammatory cytokines such as IL-6 and TNF-alpha and a causes a reduction in serum levels of C-reactive protein in obese patients or patients with metabolic syndrome [34]. In a 2016 study, obese patients under 50 years of age experienced an improvement in diastolic blood pressure after a whole-grain diet [35]. Considering the high mortality risk associated with chronic diseases such as cardiovascular disease, low-calorie diets based on whole-grain foods may reduce this risk [36].

### 3.5. M2 mRNA Markers Expression

**Figure 6:**
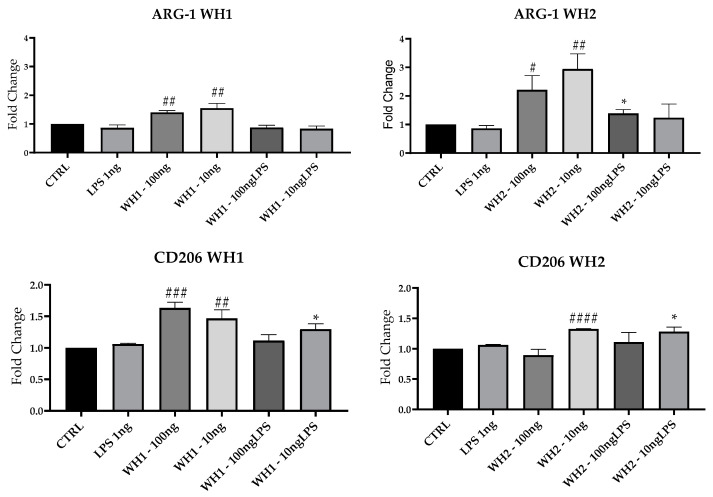

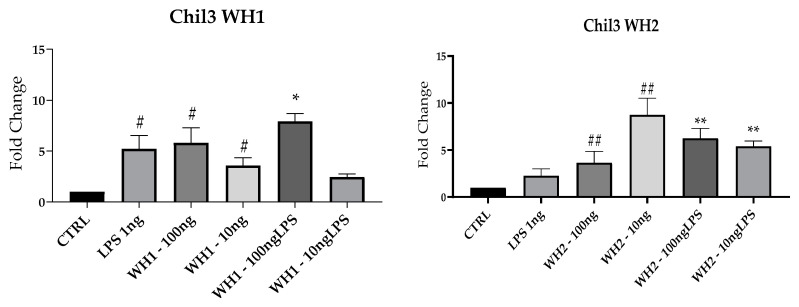
mRNA expression of ARG-1, CD206 and Chil3 evaluated by qRT-PCR. Data are shown as mean ± SD from three independent experiments performed in triplicate. Expression profiles were determined using the 2^−ΔΔCT^ method. # VS CTRL; * VS LPS; * *p* < 0.05; ** *p* < 0.01; # *p* < 0.05; ## *p* < 0.01; ### *p* < 0.001; #### *p* < 0.0001.

To evaluate the M2 status of BV-2 cells here, we performed RT-qPCR analysis and assessed mRNAs expression of Arginase-1 (Arg-1), which is associated with repair mechanisms [16]; and CD206 and Chil3 (Figure 6).

Expression of ARG-1 mRNA was induced by the addition of extracts obtained from WH1 and WH2 husk at both 100 and 10 ng/mL concentrations and compared with CTRL. Pretreatment with 10 ng/mL of WH2 husk extracts significantly increased ARG-1 mRNA expression also after the addition of LPS. The mRNA expression of CD206 increased significantly compared with CTRL after addition of WH1 100 and 10 ng/mL and WH2 10 ng/mL. In both chains, ARG-1 expression increased in cells pretreated with the extracts obtained from Pula in the presence of LPS.

Chil3 mRNA expression was induced by the extracts alone in both chains. In the presence of LPS, the WH2 extracts at the concentrations of 100 and 10 ng/mL and WH1 extracts at the concentration of 100 ng/mL stimulated Chil3 mRNA expression. 

Therefore, the extracts obtained from husk by themselves do not induce any inflammatory effect; instead, they stimulate mRNA synthesis of M2 markers, reverting microglia toward an anti-inflammatory phenotype. Neuroinflammation and oxidative stress are hallmarks of neurodegeneration, contributing to the etiopathogenesis of diseases such as AD, PD, and depression. There are still no approaches that resolve or prevent the onset of these diseases, so the identification of preventive therapeutic approaches seems urgent [37]. The ancient variety “Senatore Cappelli” has more polyphenolic components characterized by nutraceutical properties than modern varieties [38]. Polyphenols introduced through the diet manage to cross the blood–brain barrier by modulating microglia cell-mediated inflammation in neurodegenerative diseases [39]. 

### 3.6. Antioxidant Activity of SC Durum Wheat Husk

**Figure 7:**
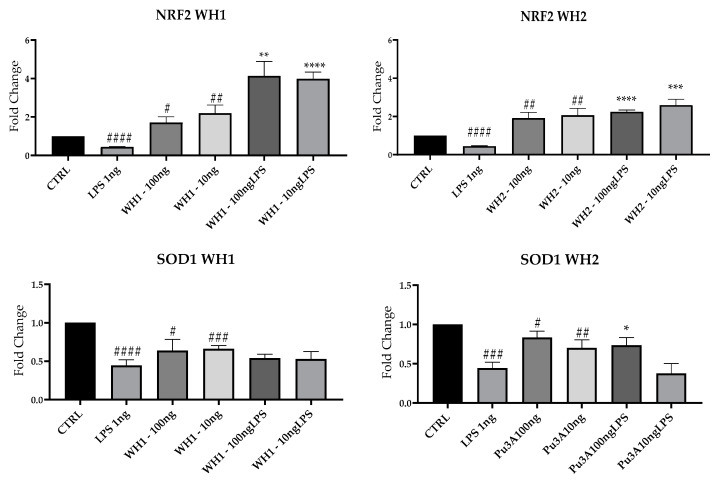
NRF2 and SOD1 mRNAs were evaluated by qRT-PCR. Data are shown as mean ± SD from three independent experiments performed in triplicate. Expression profiles were determined using the 2^−ΔΔCT^ method. # VS CTRL; * VS LPS; * *p* < 0.05; ** *p* < 0.01; *** *p* < 0.001; **** *p* < 0.0001; # *p* < 0.05; ## *p* < 0.01; ### *p* < 0.001; #### *p* < 0.0001.

Neuroinflammation underlies many neurodegenerative diseases that are incurable to date; in light of these observations, research is focusing on new therapeutic targets. The transcription factor Nrf2 regulates the expression of antioxidant genes that assist anti-inflammatory mechanisms [40]. Therefore, we evaluated by RT-PCR the mRNAs expression of NRF2 and SOD1 (superoxide dismutase1), which are enzymes protecting against oxidative stress [41]; they were significantly decreased in the presence of LPS compared with CTRL (Figure 7).

WH1 and WH2 extracts stimulated NRF2 and SOD1 mRNA expression at both 100 and 10 ng/mL, both in the absence or in the presence of LPS.

NRF2 signaling pathways could become a promising therapeutic target. Extracts of “Senatore Cappelli” wheat derivatives restore NRF2 expression related to the upregulation of SOD1, an antioxidant gene. It is known that NRF2 pathways counteract ROS production and inflammation in neurodegenerative disorders, suggesting that stimulation of NRF2 factor could play a key role as a therapeutic approach [11]. These results are in accordance with a study from 2016, where different in vitro assays showed that wheat chaff with ultrasound or hydrothermal or alkali pretreatments for enzymatic conversion showed antioxidant activity correlated with the concentration of reducing sugars [42]. 

The increase in mortality due to the rising incidence of cardiovascular diseases has focused attention on identifying wheat species with antioxidant and anti-inflammatory properties. Ancient varieties exhibit these characteristics to a greater extent than modern wheat cultivars. Studies on rats fed a diet of ancient wheat showed lower concentrations of reactive oxygen metabolites in plasma compared to rats fed a diet of modern wheat [43].

### 3.7. Life Cycle Assessment of Wheat By-Products

Life cycle assessment represents a well-established tool to measure the basis of environmental sustainability of a product’s or process’s life cycle across its entire value chain from extraction of raw materials to its disposal or recycling [11]. 

The inventory results were analyzed in order to calculate the environmental impacts for each impact category, namely GW, global warming; LU, land use; TEC, terrestrial ecotoxicity; TA, terrestrial acidification; FR, fossil resource scarcity; and WC, water consumption, as indicated in Table 5. The ReCiPe 2016 Midpoint (H) V1.05 method was used for the impact calculations.

In the wheat production system, the results were calculated for agricultural production and the milling process of 1 kg of wheat and relative impacts associated with milling wheat by-products, representing nearly 18% of the total production. The milling process has the least impact in all investigated environmental categories.

**Figure 8:**
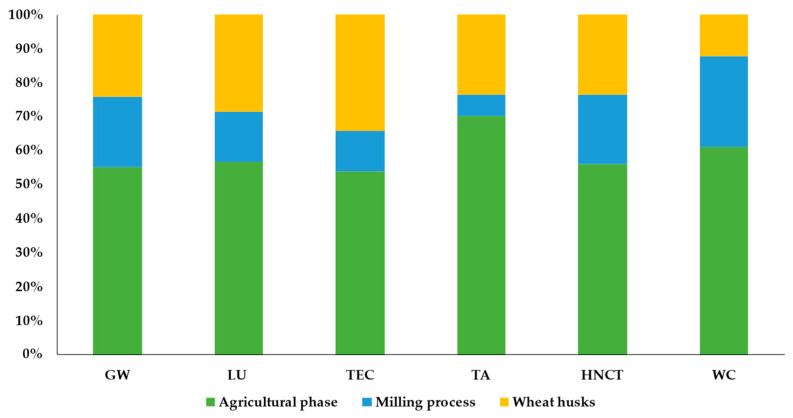
Percentage contribution (%) of each wheat process life cycle stage to environmental impact categories. GW, global warming; LU, land use; TEC, terrestrial ecotoxicity; TA, terrestrial acidification; HNCT, human non-carcinogenic toxicity; WC, water consumption.

The results showed that wheat production greatly impacts the environment, showing high values for GW (2.39 × 10^−1^ kg CO_2_ eq), LU (1.2 m^2^a crop eq), TA (2.64 × 10^−3^ kg SO_2_ eq), and WC (1.02 × 10^−2^ m^3^). Out of these values, the wheat by-products account for 23–34% of the total impacts related to the entire production process, as highlighted in Figure 8, where the results were characterized and expressed as a relative impact, where the scenario with the highest value in the impact category is set as the reference value (100), and the other is calculated accordingly. 

Principally, these impacts are mainly attributable to the emissions associated with using fertilizers from crop fields and fuel consumption for in-field operations, which contribute to 86% of the environmental impacts. According to the reviewed literature, most LCA studies on agro-food production mainly highlighted the energy-intensive production of fertilizers and the large amount applied to crop fields as the first factor responsible for climate-altering emissions [11,44,45,46], contributing most to the acidification, eutrophication, and respiratory effect categories [47].

In addition, it is worth noting that wheat production also greatly impacts human non-carcinogenic toxicity (HNCT), thus generating an amount of 4.59 × 10^−1^ kg 1.4-DCB, from which wheat husks contribute 24% of the total impacts related to human toxicity. In particular, the environmental problems causing human toxicity are mainly linked to the release of heavy metals (i.e., nickel and arsenic) and polycyclic aromatic hydrocarbons into the air [48].

#### Carbon Footprint of Alternative Scenarios for Wheat Husk System Production

Considering the environmental analysis of wheat production, it is worth noting that 25.6% of the environmental impacts in the GW category are associated with husk. As most studies highlighted, agricultural by-products are often discharged, thus creating the main disposal and environmental issue for the wheat-processing industry. In these regards, the possibility of evaluating potential scenarios for mitigating CO_2_ emissions related to wheat by-products could represent a strategic option from an environmental perspective. 

**Figure 9:**
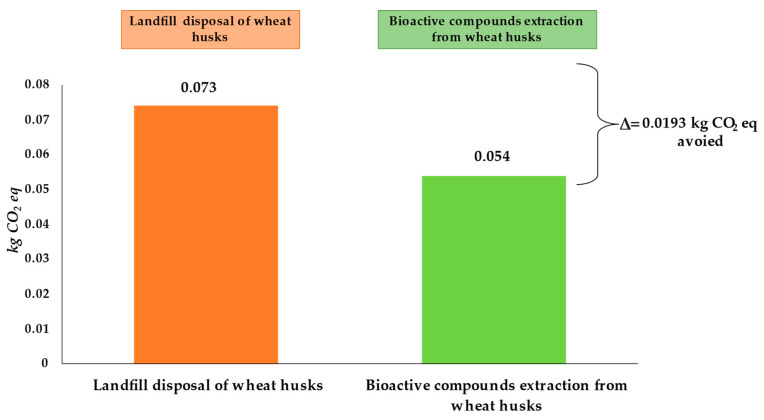
Carbon footprint of alternative scenarios for wheat husk system production.

In these regards, evaluating the potential CO_2_ emissions associated with the landfill disposal of 180 g of husk (obtained from the milling process of 1 kg of wheat), which is commonly used for animal breeding or soil conditioning purposes [10], it can be seen that it generates 0.073 kg CO_2_ eq, corresponding to 19.8%, of the total emissions generated. In the case of recycling this by-product for the extraction of high-value-added bioactive compounds, about 0.054 kg CO_2_ eq would be generated, accounting for about 14.5% of the total GHGs. Therefore, considering the possible valorization of the husk as an alternative to landfill disposal results in an avoided CO_2_ amount of 0.0193 kg CO_2_ eq, as shown in Figure 9. Different studies in the literature focused on applications possibilities for wheat milling by-products, thus focusing on their recycling processes for renewable energy production [7,44,49], biofuel and bio-gasification processes [9], as well as for the production of feedstock to produce various products including biosurfactants [50].

However, by comparing this value to the total production of durum wheat in Italy, which today amounts to about 3.5 million tons/year produced in 2022 [51], it is worth noting the possibility of avoiding nearly about 12,160 kg CO_2_ eq per year. 

## 4. Conclusions

The present work was aimed at valorizing two wheat milling husks of the ancient “Senatore Cappelli” cultivar by the recovery of bioactive compounds, thus evaluating their phenolic and antioxidant potentials and nutraceutical activity. To this purpose, a multi-methodological approach was carried out to assess both the quality and safety of raw matrices as well as the antioxidant and nutraceutical properties of wheat husk extracts.

By means of HPLC-FD analyses, wheat husk samples analyzed revealed a higher content of SER amounting to 35% of the total BAs and were confirmed to occur at BAQI values <10 mg/100 g, thus denoting no loss in the quality of analyzed samples. In addition, spectrophotometric assays showed a significant variable content in the phenolic (189.71–351.14 mg GAE/100 g) and antioxidant compounds (31.23–37.84 mg TE/100 g) within the wheat husk samples, according to the different cultivar areas of origin and the related pedoclimatic characteristics, which influence the different distribution in the content of bioactive compounds. Considering the growing interest in the renewability of food resources, this multi-methodological study builds on the potential neuroprotective role in terms of reduction of neuroinflammation and oxidative stress of waste products from sustainable agricultural supply chains.

Based on in vitro assays on BV2 cells, the two wheat husks of the ancient cultivar “Senatore Cappelli” are non-cytotoxic and increased mRNA expression of anti-inflammatory markers such as ARG-1, CD206, and Chil3. They also stimulated the expression of genes involved in the antioxidant system.

Moreover, through the application of LCA methodology, it was possible to highlight that the impacts associated with the disposal of wheat milling by-products account for approximately 9% of total wheat production. Nevertheless, the extraction of high-value-added compounds from the wheat husk can mitigate environmental and health impacts, thereby inducing 0.41% CO_2_ savings per year (12,160 kg CO_2_ eq) compared to the overall wheat-production chain. In this framework, it could be worth putting forth greater efforts in terms of energy efficiency and water productivity, thus ensuring a rationalized and sustainable production by a unit of input. This will result in long-term environmental benefits in terms of resource saving and reduced environmental pollution, which could also affect human health. 

To improve the environmental performance of wheat by-products, we mainly focused on the recovery of bioactive compounds by conventional extraction methods using hydroalcoholic solvents. Potential mitigating approaches may include the use of green solvents such as natural deep eutectic solvents (NADESs) that, due to their natural composition, can be used directly for food-fortification processes as well as for pharmaceutical and cosmetic purposes. 

## Figures and Tables

**Table 1:** Primers for Real-time PCR.

GENE	Forward Primer (5′–3′)	Reverse Primer (5′–3′)	Accession Number
mARG1	ATGTGCCCTCTGTCTTTTAGGG	GGTCTCTCACGTCATACTCTGT	NM_007482.3
miNOS	GGCAGCCTGTGAGACCTTTG	GCATTGGAAGTGAAGCGTTTC	AF427516.1
mACT-β	GGCTGTATTCCCCTCCATCG	CCAGTTGGTAACAATGCCATGT	NM_007393.5
mCOX2	AGGACTCTGCTCACGAAGGA	TGACATGGATTGGAACAGCA	NM_011198
mCCL2	GGAATGGGTCCAGACATACATTA	CTACAGAAGTGCTTGAGGTGGTT	NM_031530
mChil3	AGACTTGCGTGACTATGAAGCATTG	GCAGGTCCAAACTTCCATCCTC	NM_009892
mSOD1	GCCCGCTAAGTGCTGAGTC	AGCCCCAGAAGGATAACGGA	NM_017050
mNRF2	TCTGAGCCAGGACTACGACG	GAGGTGGTGGTGTCTCTGC	NM_031789
mCD206	TTCAGCTATTGGACGCGAGG	GAATCTGACACCAGCGGAA	NM_008625

**Table 2:** Life cycle inventory for the wheat husk system production and recycling scenarios. Inputs referred to FU: 180 g of wheat husk.

Inputs	Unit	Value	Source
**Up-stream Agricultural Process**			
Agricultural fuel	g	1.58	
Water	g	85	*EcoInvent* v3.8
Mineral superphosphate (19% P_2_O_5_)	g	0.57	
Ammonium nitrate (26% N)	g	0.78	*WFLDB*
Urea (46% N)	g	0.92	*Agribalyse* v3.0.1
Seeds	g	0.03	*EcoInvent* v3.8
Herbicide	g	0.44	*WFLDB*
Insecticide	g	3.78	
Outputs			
**Raw wheat grain**	g	1000	
**Wheat Milling Process**			
Electricity	kWh	0.06	*EcoInvent* v3.8
Outputs			
**Milled wheat grains**	g	820	
**Wheat by-products (husks)**	g	180	
**Bioactive Compounds Extraction**			
Wheat husks	g	180	
Phase 1 (Hydroalcoholic Extraction)			
Chemicals (Ethanol)	g	896	
Ultrapure water	g	210	*EcoInvent* v3.8
Electricity for ultrasonic bath	kWh	0.12	
Phase 2 (Centrifugation)			
Electricity	kWh	0.45	*EcoInvent* v3.8
Outputs			
**Wheat husk extract**	mL	885	

**Table 3:** Biogenic amines content in “Senatore Cappelli” durum wheat husks. Values are expressed as means (mg/100 g) ± standard deviation (SD) of three replicates.

Biogenic Amines Concentration (mg/100 g)	WH1	WH2
B-PEA	9.57 ± 1.33 ^b^	n.d.
PUT	1.81 ± 0.19 ^b^	0.85 ± 0.11 ^a^
CAD	0.58 ± 0.09 ^a^	2.17 ± 0.11 ^b^
HIS	n.d.	6.60 ± 0.79 ^b^
SER	11.24 ± 1.79 ^a^	17.26 ± 1.57 ^b^
TYR	n.d.	1.12 ± 0.09 ^a^
SPD	7.86 ± 0.81 ^b^	0.82 ± 0.07 ^a^
SPM	4.61 ± 0.47 ^b^	0.74 ± 0.05 ^a^
Total BAs	35.66 ^a^	43.26 ^b^
BAQI	0.42	5.46

β-PEA, β-phenylethylamine; SER, serotonin; TYR, tyramine; PUT, putrescine; CAD, cadaverine; HIS, histamine; SPD, spermidine; SPM, spermine; Total BAs, total amount of biogenic amines; B.A.Q.I., biogenic amines quality index; n.d., not detectable. The superscripts a and b in the same line denote significant (*p* < 0.05) differences.

**Table 4:** Phenolic and Antioxidant Properties of SC durum wheat husk.

	Cereal Milling Husks	
	WH1	WH2
TPC (mg GAE/100 g dw)	189.71 ± 3.97 ^a^	351.14 ± 5.91 ^b^
TFC (mg RE/100 g dw)	108.67 ± 3.44 ^b^	156.90 ± 2.31 ^a^
ABTS (mg TE/100 g dw)	31.23 ± 1.53 ^a^	37.84 ± 4.69 ^b^
DPPH (EC_50_ mg/mL)	1.45 ± 0.17 ^a^	1.34 ± 0.12 ^a^

Values are expressed as means (mg/100 g dry weight, dw) ± SD of three replicates. TPC, total polyphenols content; GAE, gallic acid equivalent; TFC, total flavonoids content; RE, rutin equivalent; TEAC, Trolox-equivalent antioxidant activity; TE, Trolox equivalent; EC_50_, wheat husk samples’ concentration providing 50% of radicals scavenging activity. The superscripts a and b in the same line denote significant (*p* < 0.05) differences.

**Table 5:** LCIA results for wheat production process compared to wheat by-products.

Impact Categories	Unit	Wheat Production	Milling Process	Wheat By-Products
Global warming	kg CO_2_ eq	2.39 × 10^−1^	9.01 × 10^−2^	1.05 × 10^−1^
Terrestrial acidification	kg SO_2_ eq	2.64 × 10^−3^	0.6	8.83 × 10^−4^
Terrestrial ecotoxicity	kg 1.4-DCB	1.79 × 10^−1^	4.02 × 10^−2^	1.14 × 10^−1^
Land use	m^2^a crop eq	1.20	2.40 × 10^−4^	3.11 × 10^−1^
Human non-carcinogenic toxicity	kg 1.4-DCB	4.59 × 10^−1^	1.68 × 10^−1^	1.93 × 10^−1^
Water consumption	m^3^	1.02 × 10^−2^	4.50 × 10^−3^	2.04 × 10^−3^

## Data Availability

Data can be accessible upon request to the corresponding author (giuliana.vinci@uniroma1.it).
